# Investigating the Relationship between Serum Level of s-Met (Soluble Hepatic Growth Factor Receptor) and Preeclampsia in the First and Second Trimesters of Pregnancy

**DOI:** 10.1155/2013/925062

**Published:** 2013-07-31

**Authors:** Farshad Naghshvar, Zhila Torabizadeh, Narges Moslemi Zadeh, Hooman Mirbaha, Parand Gheshlaghi

**Affiliations:** ^1^Department of Pathology, Mazandaran University of Medical Sciences, Sari, Iran; ^2^Department of Obstetrics and Gynecology, Mazandaran University of Medical Sciences, Sari, Iran

## Abstract

*Introduction*. Preeclampsia (PE) is an important cause of mortality and morbidity for mothers, fetuses, and the newborns. Placenta plays a pivotal role in pathogenesis of PE. Hepatic growth factor (HGF) is a cytokine expressed by the mesenchymal stalk of placental villi during pregnancy and assumes a paracrine role in trophoblasts which express its receptor (c-MET). In the present study, we investigate the diagnostic value of s-Met (the soluble form of the receptor) in the first and second trimesters of pregnancy for early diagnosis of preeclampsia. *Method and Materials*. This is a case-control study conducted on 95 pregnant women. The serum level of s-Met was measured in the first and second trimesters, and the participants were followed until delivery. 44 individuals with preeclampsia (the case group) and 51 individuals without preeclampsia (the control group) were evaluated. *Results*. Serum level of s-Met in preeclamptic participants was lower than that of the control group in both the first and the second trimesters (*P* < 0.0001). In addition, serum levels of s-Met were significantly lower during the first and second trimesters in patients with early, severe preeclampsia compared to those with late, mild preeclampsia (*P* < 0.0001). The sensitivity and specificity of s-Met in the first and second trimesters were, respectively, (83%, 94%) and (77%, 94%) for early preeclampsia and (88%, 92%) and (86%, 98%) for severe preeclampsia. *Conclusion*. Considering our findings, serum level of s-Met may be used as a predictive factor for early detection of preeclampsia. Further research is required to corroborate the functional and therapeutic value of s-Met in preeclampsia.

## 1. Introduction

Preeclampsia (PE) is a condition characterized by hypertension and proteinuria in pregnant women, where hypertension is defined as blood pressure equal to or exceeding 140/90 mmHg after the 20th week of gestation, and proteinuria is defined as either urinary excretion of more than 300 mg protein in 24 hours or presence of 3 mg/dL (≥1^+^ dipstick test) protein in two random urine samples [1]. Preeclampsia is an important cause of mortality and morbidity for mothers, fetuses, and the newborns. The pathophysiologic mechanisms leading to preeclampsia are not clearly defined; nevertheless, a cascade of events, such as abnormal invasion of the placenta, compromised placental perfusion, and placental ischemia [1], is known to play an essential role in jeopardizing the endothelial function of the mother's cardiovascular system. Placenta has a pivotal role in pathogenesis of preeclampsia, and the symptoms of the disease subside after the placenta is delivered [2]. 

It is crucial to identify women at risk of preeclampsia since these patients require close monitoring and preventive measures, such as early diagnosis and timely delivery [2]. Certain biochemical markers have been proposed for early diagnosis of preeclampsia, including placental protein 13 (pp-13) and a protein produced by the growing trophoblast (PAPPA) [3]. Another marker of interest is the hepatic growth factor (HGF) which regulates cell growth, differentiation, and morphology [1]. Moreover, HGF is a potential angiogenic factor that contributes to proliferation and migration of endothelial and smooth muscle cells and formation of new vessels in subcutaneous and retinal tissue in animal models [4]. c-Met is the receptor of the HGF, composed of an alpha chain (50 kDa) and a beta chain (140 kDa) bound through disulfide bonds. Primary c-Met may be released from the membrane of endothelial cells through proteolysis, giving rise to the soluble form (s-Met) which bonds with HGF and inhibits it from binding to its target [4]. Recent studies indicate that HGF is involved in placental growth and expansion, and inhibiting HGF production in animal models results in intrauterine demise due to placental insufficiency [4]. HGF is produced by the mesenchymal stalk of placental villi during pregnancy, and plays a paracrine role for trophoblasts which express the c-Met (receptor of the HGF). It has been demonstrated that placental HGF production is compromised in women with preeclampsia and intrauterine growth retardation (IUGR) [4]. Considering the role of s-Met in regulation of angiogenesis and other processes regulated by the HGF, it has been hypothesized that abnormal serum levels of s-Met may be beneficial in early diagnosis of preeclampsia before the clinical syndrome [4]. A study by Zeng et al. [4] reported that lowered plasma level of s-Met may be a potential biomarker for predicting severe preeclampsia early in the second trimester. One limitation of the study in [4] is the fact that it did not measure s-Met levels in the first trimester; thus, leaving unanswered questions regarding the relationship between preeclampsia and plasma levels of s-Met in the first trimester. In another study, Shin et al. concluded that elevated plasma levels of s-Met in the second trimester may be helpful for early detection of preeclampsia [5].

As preeclampsia constitutes a major cause of mortality for mothers, fetuses, and the newborns, and there is currently no appropriate screening test available for predicting it, we investigated the diagnostic value of s-Met levels in the first and second trimester of pregnancy for early detection of preeclampsia. Moreover, we compared the value of this marker in mild and severe preeclampsia.

## 2. Method and Materials

This is a prospective, nested case-control study on pregnant women referred to the healthcare centers of Sari, Iran, in 2011. The sample size consisted of 44 cases and 51 control individuals selected out of 800 pregnant women using the criteria explained after words. The inclusion criteria were pregnant women with normal blood pressure referred in the first trimester of pregnancy. The exclusion criteria were pregnant women with history of renal disease, chronic hypertension, multiple pregnancy, (overt) diabetes, and tobacco use.

After receiving information about the study, all the 800 participants gave written informed consent and completed a questionnaire. Subsequently, they were referred to the central laboratory of Sari to give 5cc blood sample in serum separated tube (SST) in the 11–13th weeks (as blood sample of the first trimester) and again in the 24–28th weeks (as blood sample of the second trimester). Serum was separated by centrifugation for 15 minutes at 1000 ×g and was preserved at −70°C.

These women were followed during the period of their pregnancy. Those developing preeclampsia were labeled as the case group and then were further classified as early or late PE, as well as mild or severe PE. The control group was selected randomly from the initial population of pregnant women without preeclampsia who were similar to those in the case group in terms of age, gestational age, body mass index, diabetes, and history of preeclampsia. 

Serum level of s-Met in both groups was measured by technicians who did not have access to the outcome of pregnancy, using CUSABIO kit to perform ELISA.

## 3. Statistical Analysis

Statistical tests for comparing the serum levels of s-Met in the above groups were completed on SPSS software. We calculated the sensitivity, specificity, positive predictive value (PPV), and negative predictive value (NPV) for this marker. Using the ROC curve, we determined the cutoff point for s-Met as the marker. Other parametric variables were analyzed using student's *t*-test, and nonparametric variables were analyzed using sum of squares. *P* values below 0.05 were considered significant. 

## 4. Results

The present study was conducted on 95 pregnant women, consisting of 44 individuals with preeclampsia (case group) and 51 individuals without preeclampsia (control group).  Table 1 presents the demographic information of the case and control groups.

The mean serum level of s-Met in women with preeclampsia was significantly lower than that of the normal women in both the first and the second trimesters (*P* < 0.0001).

The route of delivery was also significantly different in the two groups. In the case group, 31 patients (60.8%) required C-section, while it was only required for 20 cases (39.2%) in the control group.

Out of 44 cases of preeclampsia, 13 (29.5%) were early onset (<34 weeks) and 31 (70.5%) were late onset (>34 weeks). The mean serum level of s-Met in the first trimester was 177.69 ± 9.26 ng/mL in patients with early preeclampsia and 263.13 ± 28.9 ng/mL in patients with late preeclampsia. Furthermore, the mean serum level of s-Met in the second trimester was 187.79 ± 14.32 ng/mL in patients with early preeclampsia and 241.9 ± 36.18 ng/mL in patients with late preeclampsia (*P* < 0.0001 in both cases). 

**Figure 1:**
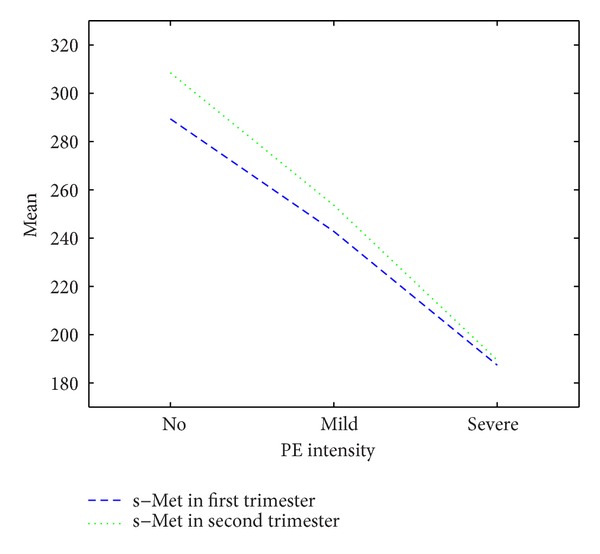
Fluctuations of s-Met level in mild and severe preeclampsia in the first and second trimesters.

Out of 44 cases of preeclampsia, 25 cases (56.8%) were mild and 19 cases (43.2%) were severe. The mean serum levels of s-Met in cases with mild preeclampsia were 242.8 ± 25.08 ng/mL and 253.60 ± 29.84 ng/mL in the first and second trimesters, respectively. For severe preeclampsia, the mean serum levels of s-Met in the first and second trimesters were 187.37 ± 22.56 ng/mL and 189.47 ± 12.68 ng/mL, respectively (*P* < 0.0001 for both cases). This is illustrated in Figure 1.

Table 2 provides the sensitivity, specificity, positive predictive value, and negative predictive value of s-Met in the first and second trimesters for mild and severe PE, as well as early- and late-onset PE. In detecting early onset PE, s-Met is more sensitive and specific in the first trimester, whereas for late onset PE, it is more sensitive and specific in the second trimester. Furthermore, in mild and severe preeclampsia, s-Met is more sensitive and specific in the second trimester. The highest sensitivity and specificity pertain to the threshold of 200 ng/mL in the second trimester for severe preeclampsia (86% and 98%, resp.). 

## 5. Discussion

In the present study, we compared the serum level of s-Met during the first and second trimesters in women who developed preeclampsia and those who did not. We demonstrated that serum levels of s-Met are significantly lower in preeclamptic pregnancies, and the cutoff points differ between the first and second trimesters. Analysis of ROC curve for data obtained from this study reveals that these threshold values are accurate and reliable for predicting preeclampsia clinically, particularly in the case of severe PE and early PE. According to our findings, patients who eventually developed PE had significantly lower s-Met levels in the first trimester of pregnancy, and these changes may occur over 3 months prior to the first clinical symptoms of hypertension or proteinuria. Moreover, we found a significant difference in s-Met levels between early- and late-onset PE in both the first and the second trimesters, with the values significantly lower for early onset PE. Similarly, severe PE had significantly lower s-Met levels in both the first and second trimesters. 

Zeng et al. [4] demonstrated that the plasma level of s-Met early in the second trimester (weeks 15–18) was significantly lower in women who developed severe PE. That research, however, did not distinguish between early- and late-onset PE, and s-Met levels in the first trimester were not addressed. In another study, Shin et al. [5] reported an elevated serum level of s-Met in the second trimester in patients with preeclampsia, which contradicts our findings and those of [4], thus highlighting the need for further research on this subject.

Early changes in s-Met production and its plasma level in women who develop PE indicate the role of s-Met in pathogenesis of preeclampsia [4]. On the other hand, despite the findings reported by us as well as [4], indicating lowered plasma level of s-Met, Iioka [6] observed no significant change in plasma HGF levels of preeclamptic patients. In another study conducted by Robinson and Johnson, the plasma HGF levels in weeks 15–20 of gestation were not significantly different between patients with severe preeclampsia and the control group [7]. Therefore, it appears that preeclampsia compromises the homeostasis between HGF and s-Met. Well-defined changes in preeclamptic patients include lowered level of vascular endothelial growth factor (VEGF) and placental growth factor (PIGF) and elevated level of pseudotyrosine kinase receptor (sFlt-1) and endoglin (sEng) which result in reduced angiogenic effects of VEGF/PIGF and abnormal function of endothelial cells in patients with PE [8, 9]. On the other hand, it appears that reduced s-Met in preeclampsia facilitates the angiogenic properties of HGF. One possibility is that certain feedbacks lower s-Met in order to prevent endothelial cells injury.

The signaling effects of HGF/c-Met are well known, especially in renal cells: inducing cell growth, reinforcing cell movement (podocytes), regeneration of epithelial renal cells, and suppression of fibrogenic cytokines in fibroblasts [10]. HGF may counter the effect of TGF-*β* and protect peritubular capillaries and endothelial cells against apoptosis [11]. Also it has been demonstrated that insufficient HGF production leads to kidney fibrosis [12]. Preeclampsia is associated with characteristic glomerular lesions, known as glomerular endotheliosis, and it appears that proteinuria in PE is the result of direct injury to renal podocytes. Thus, it is presumable that HGF/c-Met may play a role in the renal dysfunction of patients with PE. The causal relationship between lowered s-Met and endothelial and renal dysfunction in preeclampsia remains to be clarified and requires further studies.

Identifying the source of s-Met production in pregnancy may help to clarify the mechanisms disrupting serum levels of s-Met in PE. Both HGF and c-Met have been detected in human placenta, and c-Met has been localized mostly in trophoblasts and endothelial cells [12, 13].

Wajih et al. [14] demonstrated that HGF may induce the release of s-Met from smooth muscle cells and endothelial cells in human. Therefore, it is believed that HGF might be responsible for s-Met production in placenta, which may account for the elevated s-Met levels in the second trimester of normal pregnancy, as it coincides with the peak of placental HGF [15, 16]. Of course, this explanation cannot satisfactorily justify the early reduction in s-Met level in patients with PE, as reduced HGF reactivity in preeclamptic placenta has been observed only in the third trimester of pregnancy [15]. It has been documented that mother's other organs, such as liver and kidneys, may be involved in HGF and s-Met production, and abnormal s-Met levels early in pregnancy in women who will develop PE may reflect an early dysfunction in these maternal organs in the pathophysiological course of preeclampsia.

Therefore, considering the findings of this study and others, as well as the pathophysiological mechanisms of HGF/s-Met, it is possible that s-Met can serve as an appropriate marker for early detection of preeclampsia. Nonetheless, further research is required to corroborate the functional and therapeutic value of s-Met in preeclampsia.

## Figures and Tables

**Table 1:** Mean values of variables in the case and control groups.

Variable	Case	Control	*P* value
Age (years)	26.34 ± 4.98	26.15 ± 4.61	0.85
Mother's weight in the beginning of pregnancy (Kg)	78.20 ± 9.26	80.33 ± 8.48	0.24
Systolic blood pressure (mmHg)	148.18 ± 10.7	113.7 ± 4.8	<0.001
Diastolic blood pressure (mmHg)	93.86 ± 5.69	73.72 ± 4.88	<0.001
Newborn's weight (g)	3165 ± 419	3188 ± 240	0.73
s-Met of the first trimester (ng/mL)	218.8 ± 36.5	289.4 ± 54	<0.0001
s-Met of the second trimester (ng/mL)	225.9 ± 39.9	308.5 ± 55.9	<0.0001

**Table 2:** Sensitivity, specificity, positive predictive value, and negative predictive value of s-Met in the first and second trimesters for mild and severe, as well as early- and late-onset preeclampsia.

Preeclampsia	Trimester	Cutoff point	Sensitivity 95% CI	Specificity 95% CI	Positive predictive value 95% CI	Negative predictive value 95% CI	Relative risk 95% CI	Likelihood Ratio	*P* value
Early onset	1st	185	83% 51%–97%	94% 84%–98%	77% 46%–95%	96% 86%–99%	19.62 4.88–78.84	14.44	<0.0001
	2nd	195	77% 46%–95%	94% 84%–99%	77% 46%–95%	94% 84%–99%	13.08 4.19–40.8	13.08	<0.0001

Late onset	1st	250	63% 46%–78%	84% 70%–93%	77% 59%–94%	73% 58%–84%	2.82 1.73–4.58	3.97	<0.0001
	2nd	270	66% 49%–80%	86% 72%–95%	81% 62%–92%	74% 60%–86%	3.1 2.4–4.8	4.825	<0.0001

Mild	1st	260	59% 41%–75%	88% 74%–96%	80% 59%–93%	73% 58%–84%	2.91 1.70–4.74	4.941	<0.0001
	2nd	280	62% 44%–79%	89% 75%–96%	80% 59%–93%	76% 62%–87%	3.40 1.99–5.79	5.500	<0.0001

Severe	1st	195	88% 63%–98%	92% 82%–98%	79% 54%–94%	96% 86%–99%	15.14 4.8–43.8	11.69	<0.0001
	2nd	200	86% 64%–97%	98% 89%–100%	95% 74%–99%	94% 84%–99%	16.11 5.34–48.54	42.00	<0.0001
